# Prevalence of Asymptomatic Malaria among Children in the Tamale Metropolis: How Does the PfHRP2 CareStart™ RDT Perform against Microscopy?

**DOI:** 10.1155/2019/6457628

**Published:** 2019-12-21

**Authors:** Osman N. Kanwugu, Gideon K. Helegbe, Paul A. Aryee, Abass Abdul-Karim, Frank Anaba, Zulka Ziblim, Evans D. Amevi

**Affiliations:** ^1^Department of Biochemistry & Molecular Medicine, School of Medicine and Health Sciences, University for Development Studies, P. O. Box TL 1883, Tamale, Ghana; ^2^Institute of Chemical Engineering, Ural Federal University, St. Mira 28, Yekaterinburg, Russia; ^3^Department of Nutritional Sciences, School of Allied Health Sciences, University for Development Studies, P. O. Box TL 1883, Tamale, Ghana; ^4^Public Health Unit, Tamale Teaching Hospital, Tamale, Ghana; ^5^Department of Applied Chemistry & Biochemistry, University for Development Studies, P. O. Box 24, Navrongo, Ghana

## Abstract

**Background:**

Asymptomatic carriage of the malaria parasites, likewise its misdiagnosis, especially false negatives, due to the use of substandard rapid diagnosis tests (RDTs) has been shown to hinder the progress of the fight against malaria.

**Method:**

The study assessed the prevalence of asymptomatic malaria as well as the performance of *Plasmodium falciparum*-specific protein and histidine-rich protein 2 (*Pf*HRP2) CareStart™ RDT against standard microscopy in the detection of malaria infection among 345 children (1–15 yrs) from two (2) basic schools in Tamale Metropolis.

**Results:**

From the microscopy (considered as gold standard), prevalence of malaria among the asymptomatic children was found to be 2.6%, with sensitivity and specificity of CareStart™ RDT in detecting *P. falciparum* infections found to be 55.6% and 93.8%, respectively. The positive predictive value (PPV) and negative predictive value (NPV) of CareStart™ RDT were found to be 19.23% and 98.45%, respectively. There was an evidence showing a significant relation between CareStart™ RDT and microscopy in determining malaria infection (*χ*^2^ = 30.579, *p* < 0.001).

**Conclusion:**

Prevalence of asymptomatic malaria among children was found to be 2.6%. The study reported low sensitivity and PPV for *Pf*HRP2 CareStart™ RDT in an asymptomatic population at instances of low parasitaemia.

## 1. Introduction

Malaria continues to remain a global burden and a threat to world health despite increasing awareness and public heath efforts aimed at improving vector control and therapeutics and diagnostics, which in recent times have reduced the incidence of the infection worldwide [1–3]. In 2018, the World Health Organization (WHO) reported an estimated 219 million malaria cases globally with 435,000 deaths, 93% of which occurred in sub-Saharan Africa, where it is the second leading cause of death related to infectious diseases [4, 5], with pregnant women and children being the significantly affected.

Following the recent global trend, indications are that malaria in Ghana is on the decline, but the 2017 malaria report from the WHO indicated, however, an upsurge of cases in 2016. In most outpatient departments of the country, it is noted as a major cause of morbidity (40%) and mortality especially among children of school-going age as well as among children under 5 yrs (with parasite prevalence of 20.6%) [6, 7]. Ghana has been categorized as belonging to the top 15 high-burden countries with an estimated share of 4% and 3% global malaria cases and deaths, respectively [6, 8]. Malaria transmission within Ghana is heterogeneous with three (3) epidemiologically distinct strata: (i) coastal malaria with lower but perennial transmission; (ii) forest malaria characterized by moderate and perennial transmission and finally the strata within which this study was carried out; and (iii) savannah malaria with an intense but seasonal transmission [6, 9].

Asymptomatic malaria, thus *Plasmodium* infection found in nonfebrile individuals with no apparent symptoms of malaria, has been brought to the spotlight in recent times as a new challenge to the prevention and control of malaria in sub-Saharan Africa. Individuals with asymptomatic infection carry very low parasite densities for extended periods and usually go untreated. As such, they contribute to malaria transmission especially in areas with seasonal transmission by serving as *Plasmodium* reservoirs [10–12]. Identifying and eliminating these reservoirs (through chemopreventive methods like mass drug administration) therefore will play a critical role in the fight against malaria. Unfortunately, however, asymptomatic malaria especially in the northern part of Ghana is not well characterized.

Accurate diagnosis and effective and appropriate treatment of malaria play a key role in the fight against the disease. This has been well recognized in Ghana's National Malaria Control Strategic Plan for 2014–2020, as one of its objectives is to provide appropriate diagnosis to all suspected malaria cases and also prompt an effective treatment regime to 100% of confirmed malaria cases in accordance with treatment guidelines by 2020 [13]. In health centres and posts in rural and semiurban settings in Ghana where short supply of equipment, trained personnel, and electricity hinder the use of microscopy in the diagnosis of malaria, RDTs present a great potential and a useful alternative for the rapid diagnosis, for prompt and effective treatment of malaria [9, 14]. Programs such as the Foundation for Innovative New Diagnostics has enabled the WHO to make available comparative data on the performance of RDTs. However, the accuracy of RDTs and the results generated by them can still be affected by several factors including transport and storage conditions of kits and parasite density of sample, as well as epidemiology [14, 15]. Meanwhile, newer RDT types, like the ultrasensitive HRP2-based RDT (uRDT) [16], have been found to be very useful and able to detect parasites antigen in concentrations as low as 10–40 pg/ml HRP2 as opposed to 800–1000 pg/ml HRP2 by currently available RDTs. Not until such uRDTs are rolled out for screening and detection, current ones in use will remain the first choice, making it necessary for their continual assessment.

This study therefore sought to determine the prevalence of asymptomatic malaria among children in Tamale in the northern part of Ghana with seasonal transmission whilst assessing the performance of a commonly used malaria RDT in the country for its diagnoses.

## 2. Method

### 2.1. Study Area and Study Site

The study was carried out in two (2) basic schools located in Tamale Metropolis, one of the 26 districts in the northern region of Ghana. The Metropolis has >2000 inhabitants (>36% below 15 yrs.) and a total land area of 646.9 km^2^ [17]. Generally characterized as malaria endemic [18, 19], Tamale receives only one rainfall season in a year with varying daily temperatures depending on the season [20].

### 2.2. Study Design and Study Population

A school-based descriptive cross-sectional study was employed, targeting children from the ages of 1–15 yrs. Participants were selected from two basic schools with the number of participants per school determined on the basis of their willingness and availability. These two schools were randomly selected from a pool of schools in the Tamale Metropolis. A total of 345 children participated.

### 2.3. Subject Recruitment

All children aged 1–15 yrs registered with each participating school were eligible for the study, but only those who were willing and had parent/guardian consent were included. For children under 10 yrs, only those whose parents/guardian consented and also accompanied them to the registration/sample collection point were included, and for those aged between 10 and 15, an endorsed consent form from parent/guardian was enough. However, children who received antimalarial treatment within the past two weeks before the study were excluded.

### 2.4. Ethics Approval and Consent to Participate

Ethical clearance was obtained from the University for Development Studies School of School of Medicine and Health Sciences and School of Allied Health Sciences Joint Institutional Review Board. Approval was also obtained from the respective school authorities. The study, detailing purpose, benefits, and sample collection procedures, was explained to staff as well as parents in the local language (Dagbani) at a meeting, and informed consent obtained from parents before the study was initiated.

### 2.5. Data/Sample Collection

Details of each study subject such as age, sex, and place of residence were collected by oral interview of parent/guardian or the participant (>10 yrs), and a unique code for each participant was generated to label the RDT kit and microscope slide. Blood samples from finger pricks were used accordingly for both RDT and microscopy. All those involved in the blood testing process had prior clinical laboratory experience and in addition were given a week's training by an experienced and qualified laboratory technologist.

### 2.6. RDT

The rapid malaria test, PfHRP2 CareStart™ (manufactured by Access BIO, Inc., Monmouth Junction, New Jersey, USA), was used to detect *Plasmodium* species in participant samples. The RDT was based on lateral flow immunochromatography in cassette format which can detect *Plasmodium falciparum*-specific protein and histidine-rich protein 2 (HRP 2). The test was carried out on the field and read within 20 min according to the manufacturer's instructions.

### 2.7. Microscopy

Thick and thin blood smears were prepared on either ends of the same slide, air dried in the field, and transported to the Public Health Reference Laboratory at the Tamale Teaching Hospital. The slides were stained with 10% (v/v) Giemsa for 10 min and screened for the presence of plasmodial infections. The slides were read by a trained experienced laboratory technologist who was blinded to the RDT results. Parasitaemia was determined from thick blood films by counting the number of parasites per 200 white blood cells (WBCs). A slide was classified as negative if no *Plasmodium* asexual forms or gametocytes were found after counting 500 WBCs. The thin films of each specimen were subsequently examined for species identification only in those specimens in which malaria parasites were identified in the thick film. For quality control purposes, a second experienced microscopist randomly selected 5% of the slides for re-examination.

### 2.8. Data Analysis

Data were entered and analysed using the Statistical Package for Social Sciences (SPSS) version 25. Data were analysed for descriptive statistics, while results of both methods were compared using Cohen's kappa coefficient and the chi-square test. All statistical analyses were done at 95% level of significance. The sensitivity, specificity, positive predictive value (PPV), and negative predictive value (NPV) were calculated using the formulae presented below.

## 3. Results

### 3.1. Characteristics of Study Population

A total of 345 children participated in the study, of which only 31 (9.0%) were under 5 yrs and the majority (207, 60%) were from 5–10 yrs. Female participants were more (208, 60.3%) than male (Table 1). The overall mean age of participants was around 9 yrs (similar in males and females).

### 3.2. Prevalence of Malaria Infection

Using RDT, a total of 23 malaria cases were detected, while microscopy detected 9. The prevalence of *Plasmodium* infection was therefore 7.5% and 2.6% per RDT and microscopy, respectively (Table 1). All *Plasmodium* species detected in this study were *P. falciparum*. An average parasitaemia of 44.44 parasites/*μ*L (95% CI: 29.66–59.23) with a range of 16–80 parasites/*μ*L was recorded. Participants who tested positive either by RDT or microscopy were apparently healthy with no signs of malaria.

### 3.3. Demographic Characteristics Associated with *Plasmodium* Infection

The prevalence of asymptomatic *Plasmodium* infection was higher among children <5 yrs in both RDT (12.9%) and microscopy (6.5%). This, however, makes up only 15.38% and 22.22% of the total positive cases recorded by RDT and microscopy, respectively. From the results of the RDT, most asymptomatic infections were found among males (10.5%) while microscopy results indicated a higher prevalence of asymptomatic infection among females (4.3%) (Table 1). Prevalence obtained with RDT did not show any association with age (*p*=0.142) or gender (*p*=0.126). However, the prevalence by microscopy showed a gender dependency towards females (*p*=0.014) with a moderate observed power of 0.7 (<0.8, the generally accepted lower limit for consideration as statistically powerful) and a rather meagre effect size (Eta squared = 0.02).

### 3.4. Performance of RDT Compared with Microscopy

Table 2 presents a 2 × 2 contingency table of both methods, while the diagnostic performance of the RDT is presented in Table 3. *Plasmodium* infection was detected by RDT in 7.5% (26/345) of children. On the other hand, microscopy confirmed *Plasmodium* infection in only 19.2% (5/26) and the rest thus, were considered false positives. There were also 1.3% (4/319) false negatives. Overall, 98.7% of all RDT negative cases were confirmed to be negative by microscopy. There was no evidence of association between parasitaemia and RDT results (*p*=0.178). The effectiveness of the RDT in diagnosing asymptomatic malaria as presented by AUC (area under curve) of a receiver operating characteristic (ROC) curve is 0.74.

## 4. Discussion

According to the global malaria elimination program, Ghana like many other African countries is in the malaria control phase. Although several years of policy development and control interventions have led to a general decline in malaria-specific mortality, much still remains to be done if the country is to achieve malaria elimination. Detection of asymptomatic malaria and treatment of *Plasmodium* reservoirs are essential to eliminating the source of disease transmission, a crucial strategy in eradication of malaria. In Ghana, where the majority of rural and semiurban setting rely heavily on RDT due to the inability to perform microscopy, low-cost, highly sensitive, and highly specific screening tools for malaria are required.

Using microscopy in the current study, the overall prevalence of malaria was lower compared to RDT. This is consistent with recent studies by Sumari et al. [11] and Huang et al. [21] but contrary to results of a study in Myanmar where microscopy produced a higher prevalence than RDT [22]. The microscopic prevalence found in this current study is far lower than the 56% reported in 2010 and 27.5% in 2017 during a similar dry season study in the northern part of Ghana [18, 23]. This drastic reduction could probably be attributed to the numerous policies and control interventions put in place by the government and several other nongovernmental organizations. The prevalence showed by the RDT was, however, comparable to the 11.94% reported by Owusu et al. [24] in the southern part of Ghana even though the study was conducted during the rainy season during which malaria transmission is high. While the RDT presented a greater prevalence of malaria among asymptomatic males, it was, however, not significant statistically (*p*=0.126). Microscopy on the other hand did not detect any *Plasmodium* infection among males, but, however, showed a 4.3% parasite prevalence (lower than that by RDT) among females. Though there was a statistically significant association between gender and malaria in asymptomatic individuals, it is tempting to speculate that the higher prevalence observed in females according to microscopy might be a contributing factor since 20% more female than male participated in the study. Meanwhile, we were careful to avoid any bias in the sample collection; thus, study subjects were randomly recruited. Moreover, though the study demonstrated a modest statistical power, gender had a rather low effect size in relation to malaria status by microscopy. Evaluation of gender as a biological factor in asymptomatic malaria is, however, beyond the scope of this present study and therefore recommended for further studies. Nevertheless, in Myanmar, Huang et al. [21] and Zaw et al. [22] separately reported a higher parasite prevalence among asymptomatic males. Likewise, Kiemde et al. [25] found asymptomatic malaria to be prevalent among males using both microscopy and RDT. In another study conducted in Ghana, asymptomatic *Plasmodium* infection was observed to be rather prevalent among females [24]. Regardless of these discrepancies, asymptomatic malaria generally represents a significant threat to malaria control and particularly its eradication as it provides reservoirs for re-establishing the malaria transmission chain, thwarting the efforts made by malaria control strategies targeted at disrupting malaria transmission which mainly involve vector control (e.g., nationwide coverage of Indoor Residual Spraying and distribution and promotion of the use of Insecticide Treated Nets and larviciding) as well as diagnosing and treatment of symptomatic patients. It is without doubt that in order to eliminate the disease, interventions must also target asymptomatic individuals who harbour the reservoirs of the Plasmodium parasite.

Age is deemed as one of the most important factors that correlates with protective immunity in malaria endemic areas. With young children being the most vulnerable, it has been thought of that adults and older children who might have had several episodes of malaria and have acquired some form of immunity are more likely to carry asymptomatic infections [22]. In fact, several studies [26–28] have reported age-dependent asymptomatic *Plasmodium* carriage. In this study, however, contrary to a report by Touré et al. [29], individuals within the age of 5–10 recorded the lowest prevalence considering both RDT and microscopy with the highest prevalence associated with the age of <5 and >10 for RDT and microscopy, respectively. Similar studies conducted in Ghana found age range 11–15 to have the most prevalence of asymptomatic malaria [18, 24]. But, regardless of the age range with most prevalence, these studies, including this current one, reveal the presence of a “silent” reservoir of *Plasmodium* parasites which could be contributing significantly to the disease transmission. As reported by Rovira-Vallbona et al. [30], asymptomatic *P. falciparum* infections have been suggested to account for as much as approximately 30% of human-mosquito transmissions in Burkina Faso. Furthermore, unlike other studies also conducted in Ghana [1, 24, 31, 32], only one of the three known *Plasmodium* species was observed in this study.

Considering microscopy as the gold standard, the RDT generally demonstrated a moderate sensitivity far below that recommended by the WHO while on the other hand exhibiting a good specificity (>90% cf 95%) comparable with the WHO standard [33]. The sensitivity in this study though lower, is comparable to that reported by Ojurongbe et al. [34] and Osei-Yeboah et al. [1]. Also, these studies and that of Kwenti et al. [35], much like this present study, reported RDT to have a rather high specificity than sensitivity. However, contrary to this finding, several other studies have reported higher sensitivity rather than specificity for RDT in asymptomatic individuals [25, 36–38]. With regards to PPV and NPV, it was observed that RDT exhibited an almost excellent NPV with a meagre PPV. The NPV of the RDT observed by Kiemde et al. [25] and Ilombe et al. [36] is as good as that in this study, but whilst the PPV in their reports is likewise lower, it is 2–4 folds higher than that in the current study. The results of the current study however do not suggest that the PfHRP2 CareStart™ RDT is of substandard, but rather re-echo the fact that at very low parasite density (far below the 200 parasites/*μ*L minimum threshold as set by WHO) current batch of RDTs are not very useful and therefore the need for ultrasensitive RDT especially in areas where even microscopy is a challenge and PCR is simply impossible. In fact, with regards to the general performance of the PfHRP2 CareStart™ RDT, WHO in its recent round of product testing for malaria RDTs reported a 92% panel detection score (PDS) and 0.0% false-positive rate for the RDT at a minimum parasite concentration of 200 parasites/*μ*L and PDS of 100% at higher density (2000 parasites/*μ*l) [33].

Using Cohen's kappa coefficient to compare the agreement between RDT and microscopy, a fair but highly significant agreement (*k* = 0.259; *p* < 0.001) was found. Stratifying the results by age and gender did not make the agreement between the two tests any different. Other studies, however, have reported a moderate to high agreement between the two test methods [1, 25, 35, 37]. The results of the chi-square test further suggested an association between the two tests (*χ*^2^ = 30.579, d*f* = 1, *p* < 0.05). Assessing the effectiveness of the RDT to diagnose malaria in asymptomatic individuals, the receiver operating characteristic (ROC) curve showed RDT to be a good test method when compared to microscopy with area under the curve (AUC) = 0.74, similar to that reported by Osei-Yeboah et al. [1]. The discrepancies observed in this study with regards to the performance characteristics of the RDT could be ascribed to varying reasons including parasite sequestration and clonal variation *n* HRP2 expression, as well as pfhrp2 and/or pfhrp3 gene deletion since in recent studies these deletions were observed in other parts of Ghana [8, 14, 39]. Moreover, when Das et al. [16] reported that the conventional RDT is not able to detect either genes below parasite density of 25 p/*μ*L.

Although microscopy has been recommended by WHO as a gold standard in the diagnosis of symptomatic patients, many studies have, however, reported that low parasite densities especially among asymptomatic carriers are easily missed by this method due to inherent limitations such as expertise in microscopy reading, slide preparation method, staining technique, and detection limits [11, 22, 27, 40]. From a report by Wu et al. [41], it was observed that while positive results of microscopy corresponded to only 87% of RDT positive results, the RDT missed as much as 41% positive cases confirmed by PCR. This implies that though RDT seems to overdiagnose malaria cases (with lots of false positives) when microscopy is used as gold standard in particularly asymptomatic individuals, RDT could be underdiagnosing these cases if PCR was used as the reference method. In view of this, it is most likely that a higher prevalence would have been recorded if PCR was used in this study. With microscopy remaining the gold standard for malaria diagnosis in Ghana even at regional and teaching hospitals coupled with the fact that random checks of otherwise healthy people for malaria are uncommon in addition to the lack of study on a large population for asymptomatic infections in the country, the contribution of asymptomatic malaria to the overall prevalence of malaria in the country and the threat it poses to the national agenda of malaria elimination is not perceived or if at all, vaguely. It is therefore not surprising that currently the malaria control strategy of the country does not include programs targeted at the reservoir of *plasmodium* found in asymptomatic patients. Such mass drug administration (MDA), mass screening and treatment (MSAT), focal screening and treatment (FSAT), and voluntary screening and testing (VSAT), even though such programs have public health and ethical concerns, they present currently a viable way of targeting and eliminating asymptomatic infections [42]. Nonetheless, with even microscopy being a challenge to programs such as MSAT, FSAT, and VSAT, which require very sensitive methods (if the reticent reservoir of parasites is to be detected), it would be challenging for the country.

### 4.1. Limitations

Though convenient and affordable and remain the first-line diagnostic tool for malaria in Ghana and many other developing countries, microscopy is less sensitive in diagnosing asymptomatic infections. The use of microscopy as the gold standard is therefore a limitation to evaluating the performance of RDT and ultimately this current study. In addition, the use of a relatively small sample size, the scanty positive cases recorded in this study, and the fact that the RDT used only detects *P. falciparum* are further limitations in this study.

## 5. Conclusion

There could be a substantial amount of *Plasmodium* parasite reservoirs among children <16 yrs. Also, though the PfHRP2 CareStart™ has a good specificity and negative predictive value, it however, has a poor sensitivity and positive predictive value and therefore its use among asymptomatic individuals may result in missing true positive malaria cases. Efforts should therefore be made by all stakeholders in the fight against malaria to eliminate these reservoirs by improving upon the performance of current RDTs as well as investing in low-cost, highly sensitive, and specific screening tools for malaria.

## Figures and Tables

**Table 1:** Distribution of malaria prevalence in the study population according to age and gender.

Parameter		RDT *n* (%)		Microscopy *n* (%)		Total *n* (%)
		Positive	Negative	Positive	Negative	
Age range	<5	4 (12.9)	27 (87.1)	2 (6.5)	29 (93.5)	31 (9.0)
	5–10	11 (5.3)	196 (94.7)	2 (1.0)	205 (99.0)	207 (60.0)
	>10	11 (10.3)	96 (89.7)	5 (4.7)	102 (95.3)	107 (31.0)

Gender	Male	14 (10.2)	123 (89.8)	0 (0.0)	137 (100)	137 (39.7)
	Female	12 (5.8)	196 (94.2)	9 (4.3)	199 (95.7)	208 (60.3)

Total		**26 (7.5)**	**319 (92.5)**	**9 (2.6)**	**336 (97.4)**	**345 (100)**

**Table 2:** 2 × 2 contingency table.

		Microscopy		Total
		Positive (*n*)	Negative (*n*)	
RDT	Positive (*n*)	5	21	26
	Negative (*n*)	4	325	319

Total		9	336	345

**Table 3:** Performance of RDT compared to microscopy.

Characteristics		Sensitivity (%)	Specificity (%)	PPV (%)	NPV (%)	Cohen's kappa value (*p* value)	Chi-square value (*p* value)
Age range	<5	50.0	89.7	25.0	96.3	0.271 (0.106)	2.618 (<0.001^*∗*^)
	5–10	100.0	95.6	18.2	100.0	0.296 (<0.001^*∗*^)	35.984 (<0.001^*∗*^)
	>10	40.0	91.2	18.2	96.9	0.199 (0.025)	5.023 (0.025^*∗*^)
Gender	Male	0	89.8	NA	100	NA	NA
	Female	55.6	96.5	41.7	98.0	0.449 (0.139)	42.891 (<0.001^*∗*^)
Overall		55.6	93.8	19.23	98.45	0.257 (<0.001^*∗*^)	30.579 (<0.001^*∗*^)

^*∗*^Statistically significant at *p* < 0.05. NA: not applicable (because microscopy did not detect any positives in this category).

## Data Availability

All datasets on which the conclusions of the manuscript rely are attached with the manuscript.

## Supplementary Materials

Supplementary Materials Dataset of the subjects. This is an Excel document containing data transcribed from the questionnaire.
